# Biological aging acceleration in major depressive disorder: a multi-omics, multi-modal analyses

**DOI:** 10.21203/rs.3.rs-6716774/v1

**Published:** 2025-05-28

**Authors:** Breno Diniz, Shangshu Zhao, Gabin Drouard, Eero Vuoksimaa, Miina Ollikainen, Eric Lenze, Ming Xu, Richard Fortinski, George Kuchel, Jaako Kaprio, Chia-Ling Kuo

**Affiliations:** UConn School of Medicine; UConn School of Medicine; University of Helsinki; University of Helsinki; University of Helsinki; Washington University School of Medicine; University of Minnesota; UConn Health; University of Connecticut Health Center; Helsinki University; University of Connecticut Health Center

## Abstract

Major depressive disorder (MDD) is linked to a higher risk of premature aging, but the mechanisms underlying this association remain unclear. Using data from two population cohorts (UK Biobank and Finnish Twin Cohort), we evaluate the relationship between systemic and organ-specific proteomic and epigenetic aging acceleration and MDD. A lifetime history of MDD was associated with accelerated proteomic aging at both systemic and organ-specific levels—including the brain—in both cohorts, with stronger associations than those observed with systemic epigenetic aging. Systemic and brain proteomic aging acceleration were linked to higher risks of incident MDD and a greater risk of Alzheimer’s disease, related dementia, and mortality among individuals with MDD in the UK Biobank. Evidence of depressive episode remission attenuated the association between MDD and systemic and brain proteomic aging acceleration. Finally, Mendelian randomization analyses revealed a causal effect of MDD on systemic and brain proteomic aging acceleration. Our results suggest a strong bidirectional association between MDD and biological aging acceleration. Biological aging acceleration, assessed by proteomic systemic and organ-specific clocks, can serve as a novel therapeutic target for treating MDD and for mitigating the long-term risks of adverse health outcomes associated with this condition.

## Introduction

Major depressive disorder (MDD) is one of the most common mental disorders across the lifespan. Its prevalence varies in different populations, and the 12-month and lifetime prevalence estimates in the US are 10.4% and 20.6%, respectively^1^. In addition to its high prevalence, it also ranks among the five most disabling disorders worldwide^2^. Several factors contribute to the disability associated with MDD beyond the severity of psychopathology. For example, a lifetime history of MDD is associated with a higher risk of medical multimorbidity, including cardiovascular, cerebrovascular disease, and metabolic disorders, and decreased healthspan^3, 4^. MDD is also one of the most significant risk factors for mortality (including deaths by suicide), Alzheimer’s disease and related dementia (ADRD), and frailty^5–7^. Notably, these are features commonly associated with advancing chronological aging, suggesting that suffering from MDD may lead to premature aging.

The development of biological aging (BA) clocks marked a major breakthrough in understanding the mechanisms associated with the aging process^8^. BA clocks can include information from different sources, e.g., medical chemistry tests, DNA methylation, proteins; and are developed to predict surrogates of biological aging, e.g., chronological age, mortality, or changes in physiological systems function over time (pace of age)^9, 10^. Interestingly, despite different BA clocks being highly correlated with chronological age or are stronger predictors of health outcomes than chronological age, they do not systematically show a strong correlation with one another, possibly indicating that these BA clocks reflect different latent, age-related biological processes^11^.

Despite the evidence that MDD is associated with a premature aging phenotype, few studies evaluated its association with BA clocks. In a community-based study, individuals with MDD showed significantly accelerated epigenetic aging than never-depressed individuals, and a significant dose-effect with increasing symptom severity in the overall sample^12^. Another study, using a 2nd generation DNA methylation clock, the GrimAge, also showed that individuals with MDD presented with biological aging acceleration compared to non-depressed controls^13^. Another study, focusing on BA clocks derived from clinical chemistry measures (i.e., the Klemera-Doubal method Biological Aging and PhenoAge) showed that biological aging acceleration was associated with a higher risk of MDD diagnosis^14^. However, another recent study did not find significant associations between the history of MDD and biological aging acceleration measured by different DNA methylation clocks (e.g., HorvathAge, HannumAge, SkinBloodAge, PhenoAge, and GrimAge)^15^. These studies have important limitations, including relatively small sample size, cross-sectional study design, a focus on biological aging clocks derived only from DNA methylation or clinical chemistry measures, and the potential impact of antidepressant use on BA clocks and their associations with MDD. These factors limit our understanding of how MDD is related to biological aging acceleration in the general population.

To address some of the above-noted limitations from previous studies, we investigated the association between MDD and biological aging acceleration, focusing on recently developed systemic and organ-specific BA clocks based on proteomic data and DNA methylation data. We evaluated both cross-sectional and longitudinal associations between MDD and accelerated systemic and organ-specific biological aging. Next, we examined the bidirectional causal effects between MDD and biological aging acceleration using Mendelian randomization methods. Our primary hypotheses were that 1) individuals with a history of MDD show accelerated biological aging at systemic and organ-specific (e.g., brain) levels, and that 2) accelerated systemic and organ-specific biological aging predicts the incidence of MDD. We also hypothesized a bidirectional causal effect between MDD and biological aging acceleration. Further, we explored if the use of antidepressant medication is associated with an attenuation of biological aging acceleration, and if accelerated systemic and organ-specific biological aging is associated with adverse health outcomes among individuals with MDD, e.g., higher risk of ADRD and mortality. Our primary analyses were conducted using data from the UK Biobank (UKB) cohort^16^ and the findings were independently validated in the Essential Hypertension Epigenetics Study (EH-Epi) study, a sub-cohort of the Finnish Twin Cohort (FTC)^17^.

## Methods

### UK Biobank

UKB is a large population-based prospective study recruiting volunteers aged 40 to 69 years between 2006 and 2010^16^. For the current analysis, we included 53,014 participants that had proteomic data available for the calculation of systemic and organ-specific proteomic aging measures^18^. MDD diagnosis was ascertained using the first-occurrence data released by the UKB, including multi-source data based on ICD-10 codes (**Supplementary Table 1**). Patient Health Questionnaire-4 (PHQ-4) score were derived as previously reported (**Supplementary Table 1**)^14^. To identify possible cases of mild depression or that were not readily diagnosed in the UK Biobank, we included individuals with a total PHQ-4 score of ≥ 3 or lifetime MDD diagnosis (broad MDD category). Additionally, we defined a current MDD episode by PHQ-4 score ≥ 3, while remitted MDD was defined as participants with a lifetime diagnosis of MDD and current PHQ-4 score < 3. Participants without a history of MDD were classified as never depressed.

Participants with diagnosis of psychotic disorders (e.g., schizophrenia and bipolar disorder), or neurological disorders (**Supplementary Table 1**) and those with any missing baseline covariates data were excluded, resulting in a final sample of 50,297.

#### Biological Aging Proteomic Measures

The proteomic aging clock (PAC) and healthspan proteomic score (HPS) are systemic proteomic biomarkers of biological aging^19, 20^. They were developed using normalized protein expression (NPX) data from 2,920 proteins measured in the UKB with the Olink Explore 3072 assay to predict mortality and healthspan. Additionally, we included organ-specific proteomic clocks for eight tissues: brain, adipose, immune system, heart, arteries, intestine, kidneys, and liver^21^. Since higher HPS values indicate better systemic biological health, we use its inverse values (1-HPS) to facilitate the comparison with other proteomic and epigenomic aging clocks. Additional details about the calculation of the proteomic BA clocks are available in the supplementary methods.

#### Outcomes

We examined the association between a history of MDD and proteomic aging measures at baseline. We also examined the associations between PAC, HPS, and the brain proteomic aging clock with cognitive function and brain MRI image-derived phenotypes (IDPs) (**Supplementary Table 1**). Cognitive function was assessed through online cognitive tests, with measurement details available elsewhere^22^. Upon follow-up, we evaluated the association between baseline proteomic aging measures and incident MDD, incident ADRD and mortality, including deaths by suicide (**Supplementary Table 1**).

#### Covariates

We selected a priori covariates for evidence of associations with MDD and BA acceleration, including age, sex, ethnicity, education, Townsend deprivation index, body mass index, smoking status, hypertension and diabetes diagnosis status at baseline (**Supplementary Table 1**). The use of antidepressants in participants with MDD was assessed using self-reported medication data at baseline linking UKB prescription codes to ATC codes of antidepressants (**Supplementary Table 2**).

#### Statistical Methods

Linear regression models were used to examine the associations between MDD status and these proteomic aging measures at baseline, adjusting for chronological age and other covariates. Residuals from linear regression models for PAC, HPS, and the brain proteomic aging clock were correlated with cognitive function and brain MRI IDPs using Spearman correlation. Models were adjusted for chronological age and the time gap between protein measurements and the cognitive function measure or IDP.

Cox regression models were used to investigate the associations of baseline proteomic aging measures with ADRD and mortality during follow-up since recruitment. In these survival analyses, participants were censored at the date of ADRD diagnosis, death, or the last follow-up date of hospital inpatient data, whichever occurred first, and adjusted by the baseline covariates. P-values were adjusted for multiple testing using the Benjamini-Hochberg FDR method. Statistical analyses were performed using R version 4.4.3. Additional details about the statistical methods are available in the supplementary methods.

### The Essential Hypertension Epigenetics (EH-Epi) study Cohort Description

The external replication analyses were conducted in an independent sample of twins from the Finnish Twin Cohort (FTC), specifically those who participated in the Essential Hypertension Epigenetics (EH-Epi) study^17^. During in-person visits between 2013 and 2015, the twins provided fasting blood samples, clinical and physiological measurements^23^. Additional details about the EH-Epi study are available in the supplementary methods.

#### Proteomic Data

Proteomic data were obtained using the Olink Explore 3072 platform (Olink Proteomics AB, Uppsala, Sweden) from plasma samples of 415 EH-Epi twins^24^. After quality control, normalized protein expression (NPX) data were used to compute PAC, HPS, and organ-specific proteomic aging clocks for 401 twins.

### DNA Methylation Data

DNA methylation levels were measured using the Infinium Illumina HumanMethylation450K array and preprocessed with the R package ‘mefil’^25^. We used six previously generated epigenetic age estimates, available for 379 of the 401 Finnish twins^26^ (i.e., Horvath, Hannum, DNAm PhenoAge, GrimAge, the GrimAge2, and DunedinPACE).

### Depression Assessments

- Center for Epidemiological Study – Depression 20 items (CES-D-20) scale. A score of 20 or higher indicates a clinically relevant level of depression.
- Self-reported physician diagnosis from the 2011 questionnaire conducted 1–2 years before blood sampling.
- Broad MDD classification, defined as either a self-reported physician diagnosis or current use of antidepressant medication at the time of blood sampling.

### Statistical Methods

We used Generalized estimating equation (GEE) models to examine the associations between depression measures (CES-D total score, CES-D-defined MDD, and broad MDD category) and accelerated BA, as GEE models enable correction for non-independence of observations induced by family relatedness. Models were adjusted for chronological age and sex. For each BA measure, p-values were adjusted for multiple testing using the Benjamini-Hochberg FDR method, with significance assessed at the 5% level.

### Bidirectional Mendelian Randomization Analysis

Observational studies are subject to potential biases, such as unmeasured confounding and reverse causation, which can undermine causal inference. To address this issue, we conducted a bidirectional Mendelian randomization (MR) analysis to assess the causal relationship between MDD and accelerated proteomic aging. To evaluate the causal effect of proteomic aging on MDD, we selected genetic instruments associated with accelerated proteomic aging from genome-wide association studies (GWAS) on PAC, HPS, and the brain proteomic aging clock (**Supplementary Table 3**). For the causal effect of MDD on proteomic aging, genetic instruments for MDD (**Supplementary Table 4**) were selected based on a recent meta-GWAS^27^.

Two-sample Mendelian randomization (MR) analysis was conducted using the inverse variance weighting (IVW) method^28^ as the primary approach for causal inference. To assess the robustness of our findings, we performed sensitivity analyses using additional MR methods: MR-Egger regression, the Robust Adjusted Profile Score (MR-RAPS) method, and MR-PRESSO. All MR analyses were conducted using the R package *MendelianRandomization* v0.10.0. Additional details about the calculation of the Mendelian randomization methods are available in the supplementary methods.

## Results

### Cross-sectional analysis

A summary of participants (n = 50,297) with proteomic data available in the UKB is provided in **Supplementary Table 5**. Participants had a mean age of 57 years (range, 39–70), with the majority being female (54%) and of European ancestry (94%). Approximately 33% of participants held a college or university degree. The prevalence of a lifetime history of MDD was 8.9%. **Supplementary table 5** provides additional descriptive characterization of the sample included in the analysis. The correlations between systemic and organ-specific proteomic aging are shown in **Supplementary Fig. 1**.

Participants with a history of MDD (hxMDD, n = 4,477) at baseline were younger, more likely to be female, and socioeconomically disadvantaged compared to those without MDD (n = 45,820). They also had a higher prevalence of chronic diseases, such as diabetes and hypertension. Additionally, individuals with a history of MDD were more likely to have higher PHQ-4 scores at the time of recruitment compared to those without a history of MDD. Approximately 51% of participants with a history of MDD were using antidepressants at the time of recruitment (**Supplementary Table 6**).

**Figure 1.**
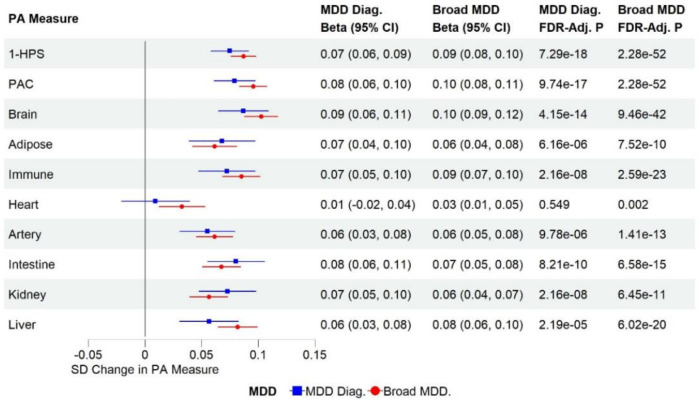
Associations between proteomic aging (PA) measures (z-scores) and a history of MDD at baseline after adjusting for covariates (MDD Diag.: diagnosed with MDD before or at baseline; Broad MDD: diagnosed with MDD before or at baseline or PHQ-4 positive at baseline)

History of MDD was significantly associated with lower HPS, higher PAC, and higher organ-specific proteomic aging measures after adjusting for chronological age and other covariates, suggesting systemic and organ-specific biological aging acceleration in MDD (Fig. 1). The associations were more robust in the broad MDD category, with the brain proteomic aging clock showing the strongest association (Fig. 1).

Additional analyses revealed that individuals with hxMDD in remission showed significantly higher HPS and PAC levels compared to individuals without a lifetime diagnosis of MDD. They also showed significantly elevated levels of multiple organ-specific biological aging clocks, most significantly in the brain, adipose, and intestine tissues, indicating that even in remission, individuals with a lifetime history of MDD may experience persistent accelerated biological aging in multiple organs that is not fully resolved even after the resolution of the depressive episode (**Supplementary Fig. 2**). Finally, individuals with hxMDD who were prescribed antidepressants also presented with significantly accelerated systemic and organ-specific biological aging (including brain proteomic aging clock) compared to those not prescribed antidepressants (**Supplementary Fig. 3**). Finally, systemic and brain proteomic aging acceleration were correlated with worse cognitive performance, particularly executive dysfunction, whole-brain and regional cortical atrophy in brain areas critical for cognitive and emotional processing, and a higher cerebrovascular burden, as measured by white matter hyperintensities, after adjusting for chronological age and time gap between blood collection and cognitive assessment or brain MRI scan (**Supplementary Figs. 4 and 5**). The full IDP analysis results are available in **Supplementary Table 7**.

#### Longitudinal analyses.

**Figure 2.**
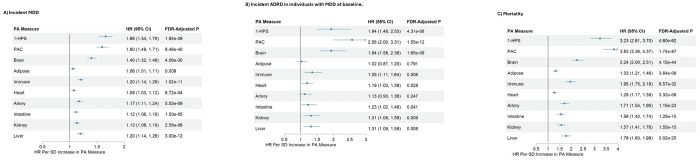
Associations between proteomic aging (PA) measures (z-scores) at baseline and incident MDD and adverse health outcomes during follow-up after adjusting for covariates

Next, we evaluated whether accelerated biological aging predicts the incidence of MDD. Among 45,820 participants without a lifetime diagnosis of MDD at baseline, 2,280 were diagnosed with MDD over a mean follow-up of 13.3 years, with a mean age at diagnosis of 63.9 years (SD = 9.6). Our analyses revealed that a lower adjusted HPS, indicating higher biological aging acceleration, was associated with a higher risk of MDD upon follow-up (a 66% higher risk per 1 SD lower HPS) (Fig. 2). Higher adjusted PAC was linked to an increased risk of incident MDD (60% higher risk per 1 SD higher PAC) (Fig. 2).

Additionally, brain biological aging acceleration conferred a greater risk of incident MDD (40% higher risk per 1 SD higher brain proteomic aging clock) (Fig. 2).

During the mean follow-up of 13.3 years, the incidence of ADRD and the mortality rate in individuals with hxMDD (n = 4,477) were 3.9% and 11.8%, respectively. Higher systemic and brain proteomic aging also predicted the risk of incident Alzheimer’s disease-related dementia (ADRD) and mortality in these individuals. More specifically, we found that a lower adjusted HPS increased the risk of ADRD and all-cause mortality by 94% and 223%, respectively (Fig. 2). Higher adjusted PAC and brain proteomic biological aging clocks also significantly increased the risks of both outcomes. Specifically, each 1 SD increase in PAC was associated with a 158% higher risk of ADRD and approximately a 283% higher risk of all-cause mortality. Similarly, each 1 SD increase in the brain proteomic aging clock corresponded to a 94% increase in ADRD risk and a 124% increase in all-cause mortality risk (Fig. 2).

### Differential impact of MDD on proteomic vs. epigenetic biological aging acceleration

Biological aging clocks have been trained using different molecular types, which may convey distinct biological information. For example, DNA methylation can be viewed as a molecular memory in cells in response to environmental influences, is relatively stable over time and transmitted with high fidelity during DNA replication^29^. On the other hand, proteins are more dynamic and proximal indicators of physiological or pathological states^30^. Using data from the EH-Epi study, we evaluated the association between depression and biological aging acceleration, based on proteomic and epigenetic clocks.

The EH-Epi study included 401 twin individuals with available proteomic data, among whom 379 also had DNA methylation data for epigenetic age estimation. Among the 401 participants, 41% were female, and 47% had never smoked. The mean age was 62.3 years (range: 56–70), with a mean BMI of 27.3 (SD = 4.9; range: 18–46). Regarding depression, the mean CES-D score was 10.1 (range: 0–47). Based on different depression definitions, 44 (11%) participants met the criteria for CES-D-defined MDD, and 63 (16%) reported a physician-diagnosed MDD. Broad MDD, defined as self-reported physician-diagnosed MDD (n = 63) and/or antidepressant use (n = 29), was identified in 75 participants (19%). Correlations between proteomic and epigenetic clocks, adjusted for chronological age, are presented in **Supplementary Figs. 6 and 7**. Correlations were stronger within proteomic or epigenetic clocks than between them, independently of chronological aging adjustments (**Supplementary Figs. 6 and 7**).

**Figure 3.**
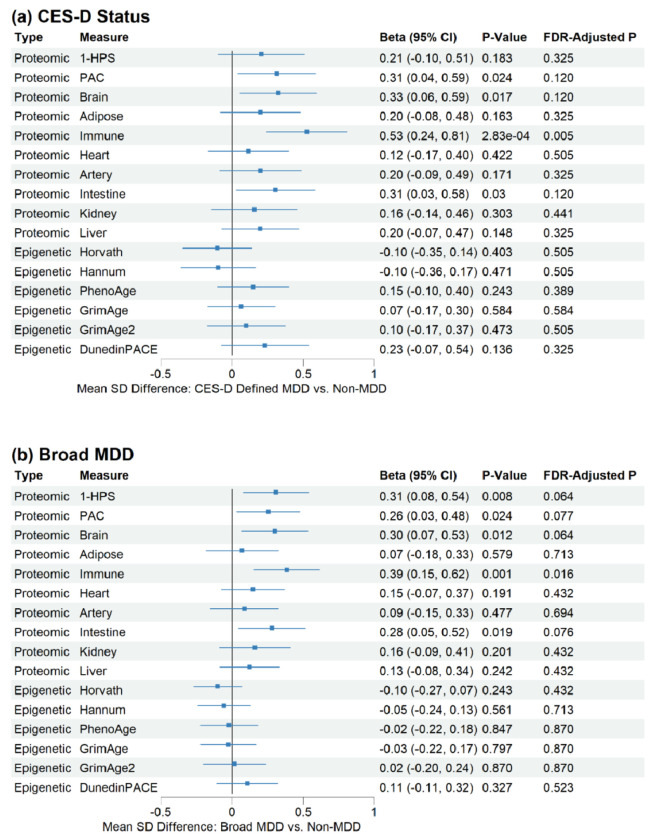
Associations of CES-D status (CES-D score ≥20 vs. <20) and broad MDD (self-reported physician diagnosis or antidepressant use vs. others) with proteomic or epigenetic aging clocks after inverse normal transformation, adjusted for chronological age and other covariates

Individuals with CES-D ≥ 20 showed evidence of biological aging acceleration based on proteomic aging measures (i.e., higher PAC, brain, immune and intestine-specific biological aging acceleration) (Fig. 3). However, CES-D status showed minimal association with epigenetic aging across all measures (Fig. 3). A sensitivity analysis using a broader MDD definition—based on self-reported physician diagnosis and/or current antidepressant use—yielded similar results (Fig. 3). These analyses independently replicate the association between depression and systemic as well as organ-specific proteomic biological aging acceleration, particularly in the brain. Moreover, they highlight the differential impact of depression on biological aging measures, with effects being more pronounced in proteomic aging clocks than in epigenetic-based measures.

### Mendelian randomization analysis

Observational studies are subject to potential biases, such as unmeasured confounding and reverse causation, which can undermine causal inference^31^. To address this issue, we conducted a bidirectional Mendelian randomization analysis to assess the causal relationship between MDD and accelerated proteomic aging.

**Figure 4.**
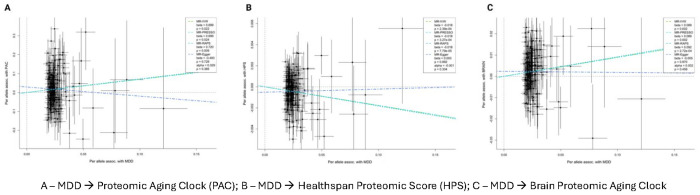
Mendelian randomization analysis for the effects of MDD on PAC, HPS, and brain proteomic aging clock

We first evaluated the causal effects of MDD on biological aging acceleration. Our findings indicate that genetically determined susceptibility to MDD is causally associated with systemic and brain proteomic aging acceleration (HPS: IWV β (mean change in HPS per one unit increase in genetically determined log odds for MDD) = −0.018, p < 0.001; PAC: IVW β = 0.699, p = 0.022; and Brain: IVW β = 0.089, p = 0.002). The results were consistent across different MR methods (Fig. 4). However, we found no significant causal effect of genetically determined proteomic aging on MDD (**Supplementary Fig. 8**). Complete MR results are provided in **Supplementary Table 8**.

## Discussion

Several mechanisms have been proposed as potential links to the development of premature aging phenotypes in MDD^32^. In the current study, systemic and organ-specific (in particular, in the brain) proteomic aging acceleration was significantly greater in individuals with a history of MDD, particularly among those showing evidence of a current depressive episode. Systemic and brain proteomic aging acceleration was associated with an increased risk of incident MDD upon follow-up, poorer cognitive performance, global and regional cortical brain atrophy, and a higher burden of cerebrovascular disease. Proteomic aging acceleration was strongly linked with incident ADRD and increased mortality risk in this population. Importantly, these findings were replicated in an independent cohort, the EH-Epi study. Lastly, Mendelian randomization analysis suggested a causal relationship between MDD, systemic, and brain proteomic aging acceleration. Overall, our study provides robust evidence of bi-directional links between MDD and accelerated biological aging, assessed through systemic and organ-specific proteomic aging measures, in middle-aged and older adults.

Prior studies have shown evidence of accelerated brain aging in MDD based on neuroimaging data, although most studies showed a small effect siz^33^, suggesting that MDD may have a small impact on brain aging, at least on the structural level. In contrast, our findings demonstrate a strong association between brain proteomic aging acceleration at baseline and a higher risk of incident MDD. They were also linked to cognitive impairment, regional brain atrophy, increased risks of ADRD and mortality, and were consistently higher in individuals with acute and remitted MDD. These findings suggest that brain proteomic aging acceleration is a potential mechanism related to the severity of MDD, poorer brain health parameters, and the long-term prognosis commonly associated with this condition.

The history of MDD is a well-established risk factor for ADRD^5^. Recent evidence does not support that MDD is associated with the build-up or acceleration of amyloid-β or Tau accumulation in the brain, the primary pathological hallmarks of ADRD^34, 35^; thus, the mechanisms linking MDD to ADRD remain elusive. Our finding that brain proteomic aging acceleration was strongly associated with a higher risk of ADRD upon follow-up can provide an alternative mechanistic explanation for such associations. From this perspective, proteomic brain aging acceleration may decrease resilience (i.e., brain reserve) against neurotoxic insults (i.e., amyloid-β deposition), thereby reducing the threshold for the manifestation of cognitive impairment and the development of dementia. Additionally, proteomic brain aging acceleration can interact with other pathological processes (e.g., cerebrovascular disease, neuroinflammation), culminating in an elevated risk of ADRD in MDD.

Our analysis revealed that individuals with remitted MDD exhibited persistent systemic and brain proteomic aging acceleration compared to never-depressed individuals, although this association was weaker than in those with an acute depressive episode. These observations may suggest that while the successful treatment of a depressive episode may attenuate its harmful effects on biological aging, it may not fully reverse them. Conversely, we found that the current use of antidepressants was associated with systemic and brain proteomic aging acceleration in both remitted and acute depressive episodes. These contradictory findings may stem from a common bias in observational studies where antidepressant use often indicates more severe depressive episode^36, 37^. These results can also provide a mechanistic explanation for previous findings showing a marginal benefit of successful antidepressant treatment in reducing mortality and the risk of dementia in MDD^38, 39^, as well as the notion that individuals with more severe depressive symptoms may be at the highest risk of developing dementi^40^.

A large body of literature suggests that peripheral systems, like the immune system, cardiovascular system, and the gut-brain axis, exert significant influence on MDD pathophysiology and long-term outcomes^41–43^. Importantly, these organs are critical for maintaining brain health and function by regulating a proper homeostatic environment, delivering energy substrates for metabolism, regulating immune function, and clearing waste^44^. Our results provide robust evidence that biological aging acceleration in multiple organs has a bidirectional association with MDD, particularly in the immune system, liver, arteries, kidneys, and intestines. These findings corroborate the perspective that MDD affects multiple peripheral organs, not just the brain, and that dysfunction in peripheral organs can increase vulnerability to MDD development. Interestingly, accelerated biological aging of adipose tissue was not strongly associated with baseline or incident MDD, despite robust epidemiological evidence for the association between MDD and obesity^45^, and the fact that obesity is one of the primary drivers of cellular senescence, a hallmark of biological aging, in this population^46, 47^.

Multiple biological aging clocks have been developed and validated using different omics modalities (e.g., DNA methylation, proteomics, metabolomics), and they may reflect distinct facets of biological aging^48^. In the independent validation cohort, we could compare the magnitude of associations between MDD, DNA methylation, and proteomic aging clocks. Our results showed that associations between MDD and biological aging were primarily observed in proteomic clocks, including the brain proteomic aging clock. These findings have important implications. First, it highlights the importance of incorporating multiple measures of biological aging to gain a deeper insight into a given condition on biological aging acceleration. This is particularly critical in clinical trials evaluating the geroscience-guided intervention effects, as a null finding may be due to the choice of surrogate measures of biological aging, rather than a true effect of the intervention. Second, our results may reflect differences between the lifelong impact of MDD on biological aging processes, as captured by epigenetic changes, versus more proximal and dynamic effects, as reflected in proteomic alterations^30^. These findings, therefore, highlight the complexity and heterogeneity of biological aging processes related to MDD, emphasizing the importance of incorporating a multi-level biological approach to gain a deeper understanding of its biological mechanisms. Further studies are needed to confirm these preliminary observations and to disentangle how biological aging clocks, whether trained on DNA methylation or proteomic data, differ in MDD.

Using findings from the most recent and largest MDD GWAS analysis^27^, we demonstrated that genetically determined MDD is causally linked with both systemic and brain proteomic aging acceleration. Taken together, our findings provide evidence that MDD is not only a risk factor but also be a primary etiological mechanism of biological aging acceleration in middle-aged and older adults. Moreover, our findings provide a putative mechanism for the recent evidence that improvement of major depressive episodes can slow cognitive decline, lower the risk of developing ADRD, and reduce mortality among individuals with MDD^39, 49^. These findings reinforce the importance of prevention, early recognition, and aggressive treatment of MDD (e.g., achieving sustained remission), how it can have a major impact on the trajectories of biological aging and mitigating the disability and long-term adverse outcomes strongly associated with this condition across the lifespan. Moreover, they provide a mechanistic explanation for the clinical and epidemiological observations that health behaviors or medical conditions associated with accelerated biological aging (e.g., obesity, smoking, sedentarism, cardiometabolic disorders)^50, 51^ are associated with a higher risk of MDD and that MDD increases the risk of multiple adverse health outcomes across the lifespan.

Our results should be interpreted considering the study’s limitations. Despite the strong validity of EHR for the identification of MDD cases in the UK Biobank^52^, the lack of formal psychiatric interviews for the majority of UK Biobank participants does not allow for a fine-grained characterization of the major depressive episode, such as currently depressed or in remission, age of onset, chronicity, the number of prior episodes, and trajectories of depressive symptoms after the diagnosis that might influence biological aging trajectories. Acute and remitted MDD were defined by the lifetime history of MDD and current PHQ-4 scores. These are crude definitions of acute and remitted depression, and we could not evaluate factors that might also influence biological aging, such as the length of remission status and the amount of prior antidepressant exposure. Likewise, we focused on the use of antidepressants (yes/no) instead of the impact of each antidepressant or class on biological aging measures. Future studies are necessary to address the fine-grained relationship between MDD and biological aging acceleration across the lifespan.

In conclusion, we provide a comprehensive analysis of the association between biological aging acceleration and MDD. Our findings support a bi-directional association between biological aging acceleration, including brain proteomic aging. We also found a robust causal effect of MDD on systemic and brain proteomic aging acceleration. Future studies should address if interventions targeting biological aging can help prevent and treat MDD, reduce disability, and improve or extend healthspan in individuals with MDD.

## Data Availability

Data access to the UK Biobank is granted upon application. The EH-Epi study data used in the analysis is available through the Biobank of the Finnish Institute for Health and Welfare (https://thl.fi/en/web/thl-biobank/forresearchers). It is available to researchers after written application and following relevant Finnish legislation. The R code for computing PAC, HPS, and organ-specific proteomic clocks and the GWAS summary statistics for proteomic aging acceleration based on PAC, HPS, and the brain proteomic aging clock can be obtained from the GitHub repository at https://github.com/kuo-lab-uchc/HPS.
